# Is Periconceptional Substance Use Associated with Unintended Pregnancy?

**DOI:** 10.1089/whr.2019.0006

**Published:** 2020-01-29

**Authors:** Lisbet S. Lundsberg, Meredith J. Pensak, Aileen M. Gariepy

**Affiliations:** Department of Obstetrics, Gynecology and Reproductive Sciences, Yale University School of Medicine, New Haven, Connecticut.

**Keywords:** alcohol, pregnancy, tobacco, unintended pregnancy, unplanned pregnancy

## Abstract

***Background:*** To evaluate the relationship between periconceptional (period before and/or after conception) substance use and unfavorable pregnancy contexts, including unintended pregnancy.

***Materials and Methods:*** This is a cross-sectional analysis of English- or Spanish-speaking women aged 16–44 years with pregnancies <24 weeks' gestation presenting to pregnancy testing clinics and enrolled between June 2014 and June 2015. Participants self-reported periconceptional substance use (tobacco, alcohol, marijuana, and other illicit substances during the 3 months before enrollment), and pregnancy “contexts,” including pregnancy intention, wantedness, planning, timing, desirability, and happiness. Multivariable logistic regression was performed adjusting for potential confounding variables.

***Results:*** We enrolled 123 women, averaging 27 ± 6 years, and mean gestational age 7.5 ± 3.0 weeks. Most participants were black, non-Hispanic (37%), or Hispanic (46%), and chose to complete the study in English (69%). Sixty-five percent participants reported use of one or more substances during prior 3 months: alcohol (54%), tobacco (31%), and marijuana (21%). In multivariate analysis, periconceptional alcohol use was associated with increased odds of unintended or ambivalent pregnancy and unwanted or mixed feelings regarding pregnancy (odds ratios [OR] = 3.29, 95% confidence interval [CI] 1.08–10.08 and OR = 2.81, 95% CI 1.07–7.36, respectively). Weekly or daily tobacco use was associated with unhappiness about pregnancy (OR = 7.56, 95% CI 1.65–34.51) and undesired or unsure pregnancy (OR = 4.00, 95% CI 1.14–14.06).

***Conclusions:*** Periconceptional alcohol or tobacco use demonstrates increased odds of specific unfavorable pregnancy contexts, including pregnancy described as undesired, unintended, unwanted, and unhappiness with pregnancy. Primary prevention of periconceptional substance use and the negative effects of alcohol and tobacco may be improved by increasing contraception access for women at risk for unfavorable pregnancy contexts.

## Introduction

In efforts to address adverse maternal and neonatal outcomes associated with substance use during pregnancy, organizations including the American College of Obstetricians and Gynecologists, Centers for Disease Control and Prevention, the Royal College of Obstetricians and Gynaecologists, and the World Health Organization have recognized the importance of addressing substance use as an important component of preconception care,^1–4^ including recommendations that pregnant women and women who *may* become pregnant abstain from any use of tobacco, alcohol, and other illicit substances.

However, focusing on preconception care in this way may be insufficient as it assumes that women have control over their reproductive lives, that contraception never fails, that all pregnancies are planned, and that such assumptions are inherently flawed.^5,6^ For example, purposeful abstinence regarding tobacco, alcohol, and illicit substance use *before* pregnancy is challenging when an estimated 45% of pregnancies in the United States^7^ and 44% of all pregnancies worldwide are unintended.^8^

Indeed, previous research has demonstrated associations between periconceptional (period before and/or after conception) substance use and unintended pregnancy,^9–11^ and pregnancy planning and timing.^12^ However, methodological weaknesses include retrospective assessment of substance use and/or pregnancy intention^11,13–16^ and lack of robust adjustment for confounders.^10^ Notably, studies frequently include live births only, thus excluding women with miscarriages or induced abortion.^11,13,15,16^ In addition to these methodologic limitations, previous studies have often narrowly focused on pregnancy intention,^9–11,14,16^ which is criticized for being overly simplistic^5,17–20^ as it may exclude other important pregnancy contexts including pregnancy wantedness, timing, desirability, and happiness with pregnancy news.^19^

Given over one-fifth of U.S. reproductive-aged women use tobacco and ∼54%–55% consume alcohol,^21,22^ periconception substance exposure may be prevalent among women with unwanted, poorly timed, undesired, or unhappy pregnancies. Including data on women with miscarriage and induced abortion, evaluating additional pregnancy contexts, and adjusting for confounders may improve understanding of the relationship between substance use and unfavorable pregnancy contexts beyond intention only and allow for innovative public health interventions.

Improved understanding may be especially relevant for addressing the potential negative effects of alcohol and tobacco given the overall prevalence of substance use in reproductive-aged women. For example, if women with periconceptional alcohol use are more likely to have pregnancies that are unintended, unwanted, unplanned, poorly timed, undesired, and/or unhappy, then primary prevention for periconceptional substance use may be improved by increasing contraception access for women at risk of these unfavorable pregnancy contexts.

We sought to examine the relationship between periconceptional tobacco, alcohol, or illicit drug use and discrete patient-centered pregnancy contexts, including intention, wantedness, planning, timing, desirability, and happiness.^19^ We hypothesized that women using substances in the periconceptional period would be more likely to report unfavorable or ambivalent pregnancy context (*e.g.*, unwanted or mixed feelings about pregnancy). Assessment of periconceptional substance use and perspectives of pregnancy context may inform efforts to improve effective contraception access and use among women at risk of unfavorable pregnancy contexts.

## Materials and Methods

### Study setting and design

We recruited women presenting for pregnancy testing at two clinical sites in New Haven, CT, from June 2014 to June 2015. A total of 225 women were approached by research staff regarding study participation. Women were eligible if they were 15–44 years of age, pregnant at <24 completed weeks gestational age, English or Spanish speaking, and completed study enrollment within 1 week of their clinic pregnancy test. However, no women under age 16 years were enrolled into the study. Recruitment and enrollment specifics have been previously published.^23^

Overall, 123 individuals were eligible, enrolled, and comprise our final sample for this study. We collected enrollment data in person using self-administered paper questionnaires, including comprehensive sociodemographic and maternal characteristics, medical conditions, and reproductive history.

### Measures of periconceptional substance use

Participants were asked about frequency of substance use during the 3 months before enrollment. Measures were obtained for tobacco use (including cigarettes, chewing tobacco, and cigars), alcohol consumption (wine, beer, and liquor), and marijuana use (pot and hash). In addition, use of cocaine, amphetamine-type stimulants, inhalants, sedatives or sleeping pills, and opioids was ascertained. Participants were asked how frequently they used each substance “in the past 3 months,” with response options including “never, once/twice, monthly, weekly, or daily.” These categories were collapsed and analyzed as three-level categorical variables of never, once/twice or monthly, and weekly or daily.

Owing to the limited number of women indicating use of other illicit substances (cocaine, inhalants, sedatives, and opioids), we analyzed dichotomous measures of illicit substance use (*other substance* use). Previous research has identified smoking and alcohol use to be related, with efforts to address these preconception behaviors jointly.^24^ Therefore, we created a summary four-level construct of alcohol and tobacco with mutually exclusive categories of use: neither tobacco or alcohol, tobacco only, alcohol only, and both tobacco and alcohol.

### Measures of pregnancy context

We assessed pregnancy context at enrollment,^19^ including “prepregnancy perceptions” of pregnancy *intention*, whether pregnancy was *wanted*, and pregnancy *planning*; *planning* was assessed using the six-item London Measure of Unplanned Pregnancy (LMUP).^25^ Context measures also included assessments of postconception perspectives: pregnancy *timing*, whether pregnancy was *desired*, and *happiness* with pregnancy news. Questions regarding pregnancy timing, intention, and wantedness come from the LMUP.

Pregnancy context was collected as three-level response measures and analyzed as two-level categorical outcome measures, with unfavorable (*e.g.*, “wrong time”) or ambivalent/neutral (*e.g.*, “okay but not quite right time”) context combined and compared with favorable (*e.g.*, “right time”) context; see Appendix Table A1. An additional component of the six-item LMUP ascertained behaviors “in preparation for becoming pregnant.”^25^ Discrete response options included whether they were taking folic acid, or ate more healthily. Responses are presented as descriptive variables but not included in multivariable modeling as they are components of planning outcome measure.

### Potential confounding variables

At enrollment, we collected sociodemographic information including age, race-ethnicity, education, employment, and relationship status. We assessed reproductive history including parity, previous miscarriage, and previous abortion. We asked participants whether they had ever been diagnosed with a chronic medical condition (*e.g.*, diabetes and thyroid problem), depression, or anxiety. Gestational age was ascertained using information from self-reported last menstrual period at time of enrollment.

### Statistical analysis

Descriptive and bivariate analyses were used to evaluate the association between substance use and pregnancy context, including chi-square and Fisher's exact tests where appropriate. Logistic regression modeling was performed to generate odds ratios (OR) and 95% confidence intervals (CIs) for the association between substance use and pregnancy contexts. Multivariable regression modeling was performed adjusting for potential confounding variables using backwards selection at *α* = 0.10. For each multivariable model, the specific substance use measure was included as well as potential confounding variables and/or independent risk factors to model the outcome of unfavorable pregnancy context (*e.g.*, wrong time/not quite right time).

Statistical analysis was performed using SAS 9.4 (SAS Institute, Cary, NC). The Yale University Human Research Protection Program and participating sites reviewed and provided approval for the study protocol. All study participants provided written consent before enrollment.

## Results

Mean age of participants was 27 (± 6) years and average gestational age at enrollment was 7.5 (± 3) weeks (Table 1).^23^ Most women were black, non-Hispanic (37%), or Hispanic (46%), parous (73%), and single or living with a partner (72%). Sixty-nine percent chose to complete the study in English and the remaining in Spanish (31%). Thirty-nine percent reported a previous miscarriage and 38% reported a previous abortion. Eighteen percent of women reported a history of a chronic medical condition (*e.g.*, asthma, diabetes, hypertension, and high cholesterol), 21% reported ever being diagnosed with depression, and 20% reported ever being diagnosed with anxiety. Only 8% of women reported taking folic acid and 19% reported eating healthier in preparation for pregnancy.

**Table 1.** Participant Demographic Characteristics (*N* = 123)

Characteristic	*n* (%)
Gestational age at enrollment	
<12 Completed weeks	114 (94.2)
≥ 12 Completed weeks	7 (5.8)
Mean gestational age in weeks (SD)	7.5 (±3.0)
Language study completed	
English	85 (69.1)
Spanish	38 (30.9)
Age	
<25	59 (48.4)
≥25	63 (51.6)
Mean age in years (SD)	26.7 (±6.3)
Range (years)	16–44
Race-ethnicity	
Black, non-Hispanic	45 (36.9)
White, non-Hispanic	13 (10.7)
Hispanic	56 (45.9)
Multiracial, other	8 (6.6)
Education	
12 Years/General Education Diploma or less	77 (63.1)
Some college, college degree	45 (36.9)
Employment	
Unemployed/homemaker	68 (55.7)
Full time/part time	54 (44.3)
Relationship status	
Single	49 (40.1)
Married	18 (14.8)
Living with partner, not married	39 (32.0)
Separated, divorced, widowed	16 (13.1)
Chronic medical problem	22 (17.9)
Ever diagnosed with depression	26 (21.1)
Ever diagnosed with anxiety	24 (19.5)
Parity	
0	33 (27.1)
1	45 (36.9)
2+	44 (36.1)
Previous miscarriage	45 (38.8)
Previous abortion	44 (37.9)
Took folic acid in preparation for pregnancy	10 (8.1)
Ate more healthily in preparation for pregnancy	23 (18.7)

Numbers may not add to *N* = 123 due to missing observations.

Most women (65%) reported periconceptional substance exposure from any source during the 3 months before enrollment (data not shown). Table 2 presents alcohol was the most common substance used (54%), followed by tobacco (31%) and marijuana (21%). Stimulant or sedative use (4.9%), and opioid (1.6%), cocaine (0.8%), or inhalant use (0.8%) were infrequent among the study sample (individual substance use data not shown). Although most participants described their pregnancy as desired (60%) and happy with pregnancy news (72%), the majority reported pregnancy as unfavorable, including unintended or unsure (67%), unwanted or mixed feelings (62%), unplanned or ambivalent (71%), and occurring at the wrong or not right time (57%).

**Table 2.** Association Between Periconceptional Substance Use and Measures of Pregnancy Context (Percentages in Table), *N* = 123

		Intention			Wantedness			Planning			Timing			Desired			Happiness		
Substance use		Intended	Unsure, unintended	*p*	Wanted	Mixed feelings, unwanted	*p*	Planned	Not sure, unplanned	*p*	Right time	Not right, wrong time	*p*	Desired	Unsure, not desired	*p*	Happy	Unhappy, neutral, do not know	*p*
Past 3 months	*N* (%)	40 (32.5)	83 (67.5)		47 (38.2)	76 (61.8)		36 (29.3)	87 (70.7)		53 (43.1)	70 (56.9)		74 (60.2)	49 (39.8)		89 (72.4)	34 (27.6)	
Tobacco				0.14			0.65			0.85			0.39			**0.02**			0.05
No	85 (69.1)	80.0	63.9		74.5	65.8		72.2	67.8		75.5	64.3		78.4	55.1		75.3	52.9	
1–2x, monthly	10 (8.1)	7.5	8.4		6.4	9.2		8.3	8.1		5.7	10.0		6.8	10.2		6.7	11.8	
Weekly, daily	28 (22.8)	12.5	27.7		19.2	25.0		19.4	24.1		18.9	25.7		14.9	34.7		18.0	35.3	
Alcohol				**0.02**			**0.01**			0.07			0.13			0.13			**<0.05**
No	56 (45.5)	62.5	37.4		61.7	35.5		61.1	39.1		54.7	38.6		51.4	36.7		46.1	44.1	
1–2x, monthly	52 (42.3)	25.0	50.6		25.5	52.6		27.8	48.3		32.1	50.0		40.5	44.9		46.1	32.4	
Weekly, daily	15 (12.2)	12.5	12.1		12.8	11.8		11.1	12.6		13.2	11.4		8.1	18.4		7.9	23.5	
Cannabis				0.24			0.21			0.83			0.21			**0.04**			0.08
No	97 (78.9)	87.5	74.7		87.2	73.7		83.3	77.0		84.9	74.3		86.5	67.4		83.2	67.7	
1–2x, monthly	14 (11.4)	5.0	14.5		6.4	14.5		8.3	12.6		5.7	15.7		6.8	18.4		7.9	20.6	
Weekly, daily	12 (9.8)	7.5	10.8		6.4	11.8		8.3	10.3		9.4	10.0		6.8	14.3		9.0	11.8	
Tobacco and alcohol				**<0.01**			**<0.01**			0.06			**0.03**			**<0.01**			**0.01**
None	50 (40.7)	52.5	34.9		51.1	34.2		50.0	36.8		45.3	37.1		44.6	34.7		40.5	41.2	
Tobacco only	6 (4.9)	10.0	2.4		10.6	1.3		11.1	2.3		9.4	1.4		6.8	2.0		5.6	2.9	
Alcohol only	35 (28.5)	27.5	28.9		23.4	31.6		22.2	31.0		30.2	27.1		33.8	20.4		34.8	11.8	
Tobacco and alcohol	32 (26.0)	10.0	33.7		14.9	32.9		16.7	29.9		15.1	34.3		14.9	42.9		19.1	44.1	
Other drug use^a^				**<0.01**			**<0.01**			**0.01**			**<0.01**			**0.02**			>0.99
No	105 (87.5)	100.0	81.5		97.8	81.1		100.0	82.3		100.0	77.9		93.1	79.2		87.2	88.2	
Yes	15 (12.5)	0.0	18.5		2.2	18.9		0.0	17.7		0.0	22.1		6.9	20.8		12.8	11.8	

Percentages presented in table as column percentages; individual percentages in categories may not total 100 due to rounding. *p*-Values based on chi-square or Fisher's exact test as appropriate. Bold text indicates a statistical significance with a *p*-value of <0.05.

^a^ Defined as use of stimulants, inhalants, sedatives, cocaine, and opioids.

Bivariate analysis demonstrates periconceptional tobacco use was associated with pregnancy desirability (*p* = 0.02). Periconceptional alcohol use was associated with measures of pregnancy intention, wantedness, and happiness (*p* < 0.05); other drug use (including stimulants, inhalants, and opioids) was associated with unfavorable or ambivalent pregnancy contexts (*p* < 0.05), except for pregnancy happiness.

Unadjusted models demonstrate increased odds of unfavorable pregnancy context with specific substance use during the periconceptional period (Table 3). Participants reporting weekly or daily tobacco use were more likely to describe pregnancy as undesired or unsure, and unhappy or neutral compared with women not using tobacco. Alcohol use once/twice or monthly was associated with unfavorable pregnancy contexts including wrong or not quite right timing, unwanted or mixed feelings, unintended or changing intentions, and unplanned or ambivalent compared with no alcohol use.

**Table 3.** Unadjusted Odds Ratio Estimates for Periconceptional Substance Use and Measures of Pregnancy Context

Substance	Unintended or intentions changing		Unwanted or mixed feelings		Unplanned or ambivalent		Wrong or not quite right time		Not desired or unsure		Unhappy, very unhappy, or neutral	
	OR	95% CI	OR	95% CI	OR	95% CI	OR	95% CI	OR	95% CI	OR	95% CI
Tobacco												
Never	Ref		Ref		Ref		Ref		Ref		Ref	
Once/twice or monthly	1.41	0.34–5.84	1.63	0.40–6.76	1.03	0.25–4.29	2.07	0.50–8.56	2.15	0.57–8.05	2.48	0.63–9.75
Weekly or daily	2.78	0.96–8.03	1.48	0.60–3.65	1.32	0.50–3.49	1.60	0.66–3.87	**3.32**	**1.37–8.05**	**2.79**	**1.12–6.95**
Alcohol												
Never	Ref		Ref		Ref		Ref		Ref		Ref	
Once/twice or monthly	**3.39**	**1.42–8.07**	**3.58**	**1.56–8.22**	**2.72**	**1.13–6.51**	**2.21**	**1.10–4.83**	1.55	0.71–3.40	0.73	0.30–1.79
Weekly or daily	1.61	0.49–5.33	1.61	0.51–5.13	1.78	0.50–6.30	1.23	0.39–3.85	3.17	0.98–10.26	3.12	0.97–10.11
Cannabis												
None	Ref		Ref		Ref		Ref		Ref		Ref	
Once/twice or monthly	3.39	0.72–16.0	2.69	0.70–10.24	1.64	0.43–6.31	3.17	0.83–12.09	**3.49**	**1.08–11.26**	**3.22**	**1.02–10.14**
Weekly or daily	1.69	0.43–6.67	2.20	0.56–8.62	1.34	0.34–5.32	1.21	0.36–4.08	2.72	0.80–9.22	1.61	0.44–5.83
Tobacco and alcohol												
None	Ref		Ref		Ref		Ref		Ref		Ref	
Tobacco only	0.36	0.06–2.16	0.19	0.02–1.70	0.28	0.05–1.69	0.19	0.02–1.70	0.39	0.04–3.59	0.51	0.06–4.80
Alcohol only	1.58	0.64–3.92	2.01	0.82–4.97	1.90	0.71–5.05	1.10	0.46–2.61	0.78	0.30–1.98	0.33	0.10–1.11
Tobacco and alcohol	**5.07**	**1.54–16.63**	**3.30**	**1.21–9.01**	2.44	0.85–7.03	**2.77**	**1.05–7.33**	**3.71**	**1.46–9.44**	2.27	0.90–5.75
Other drug use^a^												
No	Ref		Ref		Ref		Ref		Ref		Ref	
Yes	NAC		**10.50**	**1.33–82.79**	NAC		NAC		**3.53**	**1.12–11.08**	0.91	0.27–3.08

Bold text indicates a statistical significance with a *p*-value of <0.05.

^a^ Defined as use of stimulants, inhalants, sedatives, cocaine, opioids.

CI, confidence interval; NAC, not able to calculate; OR, odds ratios.

Women with combined tobacco and alcohol use demonstrated increased odds of describing pregnancy as unfavorable with respect to intention, wantedness, timing, and desire for pregnancy compared with women using neither alcohol nor tobacco. Women reporting cannabis use once/twice or monthly were more likely to report the pregnancy as undesired or unsure and unhappy or neutral feelings about the pregnancy than those with nonuse. Other substance use (stimulants, inhalants, sedatives, and opioids) was associated with unwanted or mixed feelings and undesired or unsure compared with nonuse.

In multivariable analysis (Table 4), weekly or daily tobacco use was associated with undesired or ambivalent pregnancy (OR = 4.00, 95% CI 1.14–14.06) and unhappiness or neutrality about the pregnancy (OR = 7.56, 95% CI 1.65–34.51). Women who reported once/twice or monthly alcohol use were more likely to report unintended or changing intentions regarding pregnancy (OR = 3.29, 95% CI 1.08–10.08), unwanted or having mixed feelings regarding pregnancy (OR = 2.81, 95% CI 1.07–7.36), and were less likely to report feeling unhappy or neutral regarding pregnancy (OR = 0.16, 95% CI 0.03–0.76) than women who did not drink.

**Table 4.** Adjusted Odds Ratio Estimates for Periconceptional Substance Use and Measures of Pregnancy Context

	Unintended or intentions changing		Unwanted or mixed feelings		Ambivalent or unplanned		Wrong or not quite right time		Not desired or unsure		Unhappy, very unhappy, or neutral	
Substance	OR	95% CI	OR	95% CI	OR	95% CI	OR	95% CI	OR	95% CI	OR	95% CI
Tobacco												
Never	Ref		Ref		Ref		Ref		Ref		Ref	
Once/twice or monthly	1.85	0.26–13.09	1.77	0.30–10.51	0.90	0.14–5.88	2.37	0.39–14.4	2.31	0.32–16.72	2.85	0.30–27.01
Weekly or daily	2.17	0.63–7.48	0.72	0.24–2.19	0.94	0.30–2.93	1.83	0.68–4.98	**4.00**	**1.14–14.06**	**7.56**	**1.65–34.51**
Alcohol												
Never	Ref		Ref		Ref		Ref		Ref		Ref	
Once/twice or monthly	**3.29**	**1.08–10.08**	**2.81**	**1.07–7.36**	2.15	0.80–5.79	1.71	0.67–4.36	0.52	0.13–2.12	**0.16**	**0.03–0.76**
Weekly or daily	0.68	0.13–3.50	1.20	0.33–4.35	1.24	0.31–5.02	0.57	0.14–2.30	0.69	0.09–5.45	1.33	0.21–8.36
Cannabis												
None	Ref		Ref		Ref		Ref		Ref		Ref	
Once/twice or monthly	1.99	0.35–11.35	2.06	0.49–8.73	1.33	0.31–5.71	2.23	0.52–9.60	1.38	0.29–6.56	3.45	0.65–18.20
Weekly or daily	1.42	0.27–7.41	1.96	0.45–8.54	1.66	0.39–6.97	1.53	0.40–5.78	1.69	0.33–8.72	3.26	0.47–22.66
Tobacco and alcohol												
None	Ref		Ref		Ref		Ref		Ref		Ref	
Tobacco only	0.20	0.02–1.84	0.10	0.01–1.21	0.18	0.02–1.62	0.24	0.02–2.80	0.23	0.01–5.33	0.82	0.06–11.33
Alcohol only	0.86	0.26–2.93	1.33	0.42–4.23	1.04	0.30–3.61	0.59	0.20–1.78	**0.09**	**0.01–0.55**	**0.07**	**0.01–0.48**
Tobacco and alcohol	3.50	0.84–14.59	1.92	0.54–6.86	1.61	0.43–6.01	2.19	0.72–6.61	2.41	0.62–9.28	2.91	0.75–11.26
Other drug use^a^												
No	Ref		Ref		Ref		Ref		Ref		Ref	
Yes	NAC		6.91	0.83–57.33	NAC		NAC		1.30	0.27–6.30	0.18	0.03–1.00

Bold text indicates a statistical significance with a *p*-value of <0.05; Variables including employment, age, gestational age, education, marital status, race/ethnicity, parity, chronic medical problem, language, previous abortion, previous miscarriage, history of depression, history of anxiety, other substance use in addition to exposure of interest, and recruitment site evaluated as potential confounders in multivariable modeling using backwards selection.

^a^ Defined as use of stimulants, inhalants, sedatives, cocaine, and opioids.

NAC, not able to calculate.

Use of alcohol (not with tobacco use) demonstrated reduced odds for undesired or ambivalent, and unhappy or neutral feelings compared with those with neither alcohol nor tobacco use. No significant association was observed between cannabis use or other substance use and unfavorable pregnancy contexts.

## Discussion

Among a diverse cohort of women enrolled in early pregnancy, periconceptional substance use demonstrated increased odds of unfavorable pregnancy contexts. Tobacco use was associated with increased odds of undesired or unsure pregnancy, and unhappiness or neutrality regarding the pregnancy, but was not associated with pregnancy intention, wantedness, planning, or timing. Alcohol use was associated with increased odds of pregnancy described as unintended or intentions changing, unwanted or mixed feelings, and unplanned or ambivalent, but was not associated with pregnancy planning, or desirability. Marijuana use did not show a significant association with any pregnancy context, and periconceptional substance use was not associated with pregnancies that were unplanned or ambivalent, or that occurred at the wrong or not quite right time.

Previous research has demonstrated an increased risk of alcohol use and unintended pregnancy,^11,26,27^ although alcohol exposure is often assessed as patterns of heavy or binge drinking.^11,27^ Studies have also reported an association between preconception and prenatal smoking and unwanted pregnancy^15,28^; however, we observed no association with tobacco smoking and unwanted pregnancy but rather an increased likelihood of undesired or ambivalent pregnancy, and unhappiness with pregnancy news among women smoking tobacco during the periconceptional period.

Our robust analysis of multiple periconception substances and unique measures of pregnancy context in early pregnancy improves upon the current literature and illustrates the complexity of these relationships, which has important public health implications. For example, despite frequent public health focus on *unintended* pregnancy and substance use,^9–11^ we demonstrate substance use was also associated with unwanted, undesired, and unhappy pregnancies. Public health efforts focused solely on pregnancy intention would potentially miss these other important pregnancy contexts, especially pregnancy contexts that reflect women's perspectives about the pregnancy after its diagnosis (desirability and happiness).

Our study improves upon earlier studies that are limited by potential misclassification of pregnancy intention based on complicated algorithms,^13,16^ utilize national surveys^11,13,16^ with retrospective assessments of pregnancy context and substance exposure that may be subject to recall bias,^11,14,15,26^ and lack robust adjustment for potential confounders.^10^ In addition, our study is strengthened by enrolling women early in gestation; assessing substance exposure during the 3 months before enrollment (prospective to pregnancy outcome); directly assessing unique pregnancy contexts beyond intention, thus minimizing potential recall bias; and adjustment for multiple covariates in statistical analysis.

After adjustment for potential confounders including history of depression and/or anxiety, most significant substance use risk estimates were attenuated, demonstrating the importance of comprehensive multivariable modeling that includes evaluating history of depression and/or anxiety. Finally, we recruited women presenting for pregnancy testing, inclusive of all pregnancy outcomes including induced abortion, thus enhancing generalizability of our findings compared with studies frequently restricted to live births only.^11,13,16,26^

Another strength of our study is its potential external generalizability. Our cohort is diverse in participant characteristics including race-ethnicity, marital status, education, and language. Research among a sociodemographically diverse population provides opportunities to better understand potential health disparities.

Periconceptional substance use in this study is similar to or above national estimates.^29^ Specifically, reported alcohol use (54%) is consistent with previous research among reproductive aged women,^21,22,29^ whereas tobacco and marijuana use in our study (31% and 21%, respectively) are above estimates among U.S. reproductive-aged women,^21^ suggesting reporting bias may be minimal. Notably, marijuana use is within estimates of use among younger urban populations (15%–28%).^30^ Illicit substance use in our study, including marijuana (21%), cocaine (<1%), inhalants (<1%), and opioids (1.6%), is collectively higher (22.8%) than composite illicit drug use among pregnant (5.4%) and nonpregnant (11.4%) U.S. women of age 15–44 years,^29^ and greater than previously reported periconceptional illicit substance use.^31^

Several study limitations are important to acknowledge. We did not assess income or recent contraception use, which may be important potential confounders. Our relatively small sample limits further stratification of substance exposure, and we are limited in our assessment of high levels of substance exposure. Specific substance measures, such as alcohol frequency, are limited in that they may not fully capture the scope of substance use and behavior. Further research delineating patterns of substance use (*e.g.*, binge drinking) and higher sustained levels of substance use (*e.g.*, chronic heavy drinking) and unfavorable pregnancy contexts would be informative.

In addition, few women in our study report using other illicit substances, including opioids; these substances are analyzed as a composite measure, precluding evaluation of individual illicit substances, or differentiation between illicit versus medically prescribed substances. Although self-reported substance use is subject to recall and social desirability bias, periconceptional exposure is assessed early in pregnancy among women averaging 7.5 weeks' gestation (range 3.7–18.7 weeks). Although exposure during the “past 3 months” may vary and encompass pre- and postconceptional period for most participants, methodological consistency was applied assessing exposure within 3 months before enrollment and pregnancy diagnosis, thus minimizing bias.

Further research is warranted identifying detailed substance exposure during the specific immediate preconception period as well as postconception period before pregnancy recognition, compared with after pregnancy recognition when behavior is often modified. Biomarkers of exposure were not collected for our analysis, which would increase precision of exposure assessment. In addition, although underreporting may threaten exposure assessment, acceptable agreement between self-reported substance use and toxicology results has been reported.^32^

Our study demonstrates associations between periconceptional substance exposure and unique measures of pregnancy context among a diverse cohort of pregnant women. These findings support the need for more nuanced public health interventions that improve upon overly cautious and potentially impractical advice to *all* reproductive-aged women to avoid substances in case they get pregnant.^33,34^ Instead, public health efforts that support women's “reproductive life planning” and encourage patient-centered assessments^6^ regarding potential pregnancy contexts, identify those at risk of unfavorable pregnancy contexts (*e.g.*, unintended, unwanted, and undesired), and facilitate contraceptive access for those who want to avoid pregnancy may be more beneficial.

More specifically, our finding that periconceptional alcohol use was associated with increased odds of unintended or ambivalent pregnancy and unwanted or mixed feelings regarding pregnancy provides potential opportunities for decreasing negative birth outcomes associated with alcohol-exposed pregnancies. Furthermore, enhancement of preconception education and delivery of evidence-based information are needed, along with interventions to optimize preconception health.^35^

Specific interventions to address preconceptional alcohol and tobacco use and effective contraception for those at risk of unintended pregnancy have been demonstrated^24^ and may be an important step to address the increased risk of unfavorable pregnancy context measures we observe among women with periconceptional substance use. For example, the CHOICES Plus trial demonstrated a preconception intervention reduced the risk of alcohol- and tobacco-exposed pregnancies within primary health care settings,^24^ and future efforts integrating similar approaches within reproductive health care and family planning settings should be evaluated.

Notably, our findings also reinforce growing literature that asserts pregnancy planning and intention may not have relevance for some women,^5,18^ and warrant further assessment of improved measures regarding pregnancy contexts,^5,17–20^ including how, when, and where these expanded context measures can be best used in clinical practice.

Our study highlights important pregnancy context measures beyond the construct of intention, and demonstrates opportunities to enhance education regarding contraception, substance use, and preconception health among reproductive-age women, especially those with the potential for unintended, unwanted, undesired, or unhappy pregnancies. These findings underscore the potential for increased contraceptive access as a public health primary prevention strategy for substance use in pregnancy, particularly for women at risk for unfavorable pregnancy contexts.

## Abbreviations Used

CI: confidence interval
LMUP: London Measure of Unplanned Pregnancy
NAC: not able to calculate
OR: odds ratios

## Appendix Table A1

**Appendix Table A1.** Measures of Pregnancy Context

Context	Enrollment question and response	Context measure for analysis
Prepregnancy perspectives of pregnancy: intention, wantedness, planning		
Intention^a^	Just before I became pregnant I intended to get pregnant	Intended
	Just before I became pregnant my intentions kept changing/… I did not intend to get pregnant	Unintended or intentions changing
Wanted^a^	Just before I became pregnant I wanted to have a baby	Wanted
	Just before I became pregnant I had mixed feelings about having a baby/… I did not want to have a baby	Unwanted or mixed feelings
Planning^b^	Planned. (scoring 10–12 on LMUP)	Planned
	Ambivalent (scoring 4–9 on LMUP) unplanned (scoring 0–3 on LMUP)	Unplanned or ambivalent
Postconception perspectives of pregnancy: timing, desired, happiness		
Timing^a^	In terms of becoming a mother (first time or again), I feel that my pregnancy happened at the right time	Well-timed
	In terms of becoming a mother (first time or again), I feel that my pregnancy happened at the okay but not quite right time/…my pregnancy happened at the wrong time	Not quite right time or poorly timed
Desired	Is this pregnancy desired?—Yes	Desired
	Is this pregnancy desired?—Not sure/Is this pregnancy desired?—No	Undesired or not sure
Happiness	Rate how happy you felt when you found out you were pregnant: Very happy, somewhat happy	Happy
	Rate how happy you felt when you found out you were pregnant: very unhappy, somewhat unhappy, neutral, do not know	Unhappy, neutral

Table of context measures adapted from Gariepy et al.^19^ and Lundsberg et al.^23^

^a^ Pregnancy intention, wantedness, and timing based on questions from the LMUP.^25^

^b^ Planning based on the LMUP.^25^

LMUP, London Measure of Unplanned Pregnancy.
